# A meta-analysis of technology-based interventions on treatment adherence and treatment success among TBC patients

**DOI:** 10.1371/journal.pone.0312001

**Published:** 2024-12-02

**Authors:** Mega Hasanul Huda, Muhamad Fauzi Rahman, Yusuf Zalaya, Muhammad Amirul Mukminin, Telly Purnamasari, Harimat Hendarwan, Amir Su’udi, Armedy Ronny Hasugian, Yuyun Yuniar, Rini Sasanti Handayani, Rudi Hendro Putranto, Aris Yulianto, Anton Suryatma, Mieska Despitasari, Riswal Nafi Siregar

**Affiliations:** 1 Faculty of Nursing, University of Indonesia, Depok, West Java, Indonesia; 2 Research Center for Pre-Clinical and Clinical Medicine, National Research and Innovation Agency, Cibinong Science Center, Cibinong, West Java, Indonesia; 3 College of Public Health, Taipei Medical University, New Taipei City, Taiwan; 4 Faculty of Public Health, University of Indonesia, Depok, West Java, Indonesia; 5 School of Nursing, College of Nursing, Taipei Medical University, New Taipei City, Taiwan; King Faisal University, SAUDI ARABIA

## Abstract

Various technology-based interventions have been designed to improve medication adherence and treatment success. However, research on the most effective mode to address this issue is still limited. Our study evaluated the effectiveness of technology-based interventions in improving treatment adherence, completion, and treatment success among tuberculosis (TBC) patients. We conducted a meta-analysis of randomized controlled trials by searching articles from six databases including PubMed, Science Direct, Cochrane, Jstor, Embase, and Scopus from 2018 to April 2023. Two independent reviewers assessed the study quality using the Cochrane Risk of Bias 2.0 tool. We analysed the data using a random-effects model. We also conducted publication bias and sensitivity analysis. In total, 13 studies were identified and 4,794 participants were included in the meta-analysis. The results indicated that technology-based interventions were effective in improving treatment adherence, completion, and success (Odds Ratio (OR): 2.57, 95% Confident Interval (CI): 1.01–6.50, I^2^ = 86.6%; OR: 1.77, 95% CI: 0.95–3.28, I^2^: 82.3%; OR: 1.61, 95% CI: 0.85–3.06, I^2^: 84%, respectively). We examined the possibility of publication bias in the published studies included in this systematic review. However, no evidence of publication bias was found. From the sensitivity analysis by removing one study randomly, we found that our results are robust. Based on the results, we can conclude that technology-based interventions like MERM, text-based messages, video conferencing, and VOT are effective in increasing treatment adherence and completion in tuberculosis management. Therefore, technology shows immense potential in enhancing patient outcomes.

## Introduction

Tuberculosis (TB) is an infectious disease caused by *Mycobacterium tuberculosis*. More disorders affecting these parts of the lungs continue to endanger the global population’s health [1]. Successive increases in the global tuberculosis incidence in 2021 and 2022 indicate that by 2022, the tuberculosis incidence rate had returned to the level observed in 2019. When examining trends globally, the net decline in the tuberculosis incidence rate from 2015 to 2022 was 8.7%—significantly short of the World Health Organization’s goal within their End TB Strategy of a 50% reduction in incidence by 2025 [2]. Patients with TB are spread across countries; however, the majority are located in medium- and low-income countries. Bangladesh, China, India, Indonesia, Nigeria, Pakistan, the Philippines, and South Africa account for less than half of all TB patients [3].

Close collaboration between healthcare providers and TB patients is required for effective treatment programs. Medication adherence of TB patients is a critical component of the treatment [4]. The medication adherence rate of TB patients is reported to be 40% in developing countries [5], reflecting a relatively poor success rate of treatment compared to the WHO target of at least 85–90% TB treatment success rate which should be achieved for all case diagnoses. A poor medication adherence rate can result in treatment failure, MDR TB, prolonged infection, and poor treatment outcomes [6]. In various countries, several treatments have been developed and proved to be effective in increasing the medication adherence of TB patients and achieving positive results in active TB patients [7]. Interventions include those employing technology [8] as well as those that do not [9–11].

Previous systematic reviews and meta-analyses indicate that directly observed therapy (DOT) provides no advantage over self-administered treatment (SAT) in preventing relapse or adverse drug reactions [12]. Additionally, evidence shows that DOT is more appropriate for use in a community context where people live with extended families rather than in an urban setting [13]. Given these evidence-based findings, a holistic public health approach addressing the multifaceted influences on tuberculosis treatment outcomes, rather than a singular focus on observation alone, could optimize results.

Another intervention developed in Senegal is a counseling and decentralized treatment package in which patients may select DOT supporters and activities that might improve the outcomes. This intervention is appropriate for nations with limited resources, but since it is a package, the effectiveness of each intervention within the package cannot be assessed [10]. Another example is a monthly TB voucher initiative in South Africa. This intervention is a type of financial assistance given to TB patients during the treatment. However, further study is needed to determine the best strategies to ensure consistent and appropriate support for those eligible [11]. There are also other alternative of interventions, such as nutritional supplements for TB patients in East Timor. However, this intervention can only increase the patients’ weight and has no effect on medication adherence [14].

With the numerous shortcomings of such non-technology interventions, technology-based interventions have been developed. Technology-based interventions can assist in reminding the patients about medication-related issues, facilitating digital observation while taking the medicine, and determining the patients’ dose history and triage, depending on the degree of compliance–all of which can aid in the provision of individual TB treatment with varied levels of risks [15]. The technology-based interventions employ shared methods such as phone-based technology, monitoring devices, smartphone-based technologies, digital pill boxes, and ingestible sensors that provide a patient-centric approach to increase TB medication adherence [7]. Significant forms of technology-based interventions influencing TB medication adherence include the use of Medication Event Reminder Monitor (MERM) in Peru [16], 99DOTS low-cost, mobile phone-based technology in India [17], SMS and electronic pill boxes in China [8], the use of VDOT in Mexico [18] and ingestible sensors in the United States [19]. However, other technology-based interventions, such as SMS reminders [20] and 99DOTS in Uganda [21], are less significant in terms of TB medication adherence.

Evidence against the accuracy and clinical effectiveness of technology-based interventions on TB medication adherence was still required. This is due to the results of inconsistent technology-based interventions, hence combining randomized controlled trial data through meta-analytic pooling can significantly increase overall sample size [22]. Additionally, pooling is hypothesized to provide higher-quality evidence via strengthening generalizability, reproducibility, and precision of results [23]. For this reason, a study with a systematic review design and meta-analysis on the effect of technology-based interventions on medication adherence in TB patients needs to be conducted. The aim of this study is to examine the effectiveness of technology-based interventions on medication adherence and treatment success among TB patients through a systematic review and meta-analysis.

## Methods

### Design

A systematic review and meta-analysis study is reported in accordance with the PRISMA statement guidance [24]. The research protocol was registered with PROSPERO under registration number CRD42023414741 prior to conducting the study. Researchers conducted preliminary searches, but no existing or ongoing studies with similar topics to this research were found.

### Sample

#### Eligibility criteria

This study only includes primary studies with Randomized Control Trial (RCT) designs, with the following inclusion criteria: 1) Participants are tuberculosis patients aged 18 years or older, confirmed as having pulmonary tuberculosis through microscopic sputum examination with clinical signs; 2) Participants are receiving tuberculosis treatment; 3) The intervention involves technology-based support, reminders, or monitoring; 4) The control group receives support from a family member referred to as Directly Observed Therapy (DOT); 5) Studies report outcomes related to the proportion of treatment adherence, success, and completion; 6) Articles are published within the last 5 years and written in English.

### Search strategy

A comprehensive literature search was conducted using a combination of keywords, including tuberculosis OR pulmonary tuberculosis OR sputum-positive tuberculosis AND adherence OR compliance OR concordance OR treatment OR anti-tuberculosis medication OR intervention OR therapy OR treatment completion OR completion rate AND health education OR technology-based intervention OR Telephone OR cellular phone OR wireless technology OR reminder system OR text OR message OR Phone text OR mobile application OR voice call OR MMS OR digital OR website OR m-Health OR Mobile Health OR tele-Counselling OR teleconference OR Video OR educational technology OR Instructional Technology. The literature search was performed across six electronic databases: PubMed, Science Direct, Cochrane, Jstor, Embase, and Scopus, spanning from 2018 to April 2023. Articles with technology-based interventions and outcomes related to treatment adherence and treatment success were included. Manual cross-referencing of relevant articles was also carried out. We describe the detailed search strategy for each database in S1 Table.

All studies meeting the criteria were selected, and duplicate studies were removed. Titles and abstracts of the chosen articles were screened. Three independent reviewers assessed all eligible studies, and any differences of opinion were resolved through discussion. Three authors independently conducted data extraction using a structured data extraction form, which included: participant characteristics (sample size, age, and education), intervention and objectives, intervention type (mode, frequency, duration, content, phases), intervention providers, intervention outcomes, and outcome measures (research outcomes and outcome assessment tools).

### Outcome

The study outcomes were defined as follows:

1. Treatment success is defined as patients with a final diagnosis of cured or treatment completed, whereas treatment failure includes patients lost to follow-up or with clinical failure [1, 25].
2. Treatment adherence was measured in terms of the proportion of missed doses, the proportion of patients who missed at least one of the total doses scheduled at the time of inclusion in the study, and the proportion of patients who missed more than 10% of doses.
3. Treatment completion: receiving at least 11 doses within 16 weeks [25].

### Risk of bias assessment of included studies

Two reviewers independently screened the studies and assessed their quality using the Cochrane Risk of Bias 2.0 (RoB 2.0) tool. This assessment included the evaluation of the following domains: randomization process, deviations from intended interventions, missing outcome data, outcome measurement, selection of reported outcomes, and overall bias. Each domain was categorized as low risk of bias, high risk of bias, or requiring some concern [26]. Specifically, studies with a low risk of bias were identified when they appeared to be free from potential sources of bias, while studies with a high risk of bias were identified when at least one major bias risk was detected. Some concern was described as the presence of bias risk arising from either inadequate information about bias or insufficient reasons [26]. Any differences in outcomes among the researchers were resolved through discussion until a consensus was reached.

### Statistical analysis

The analyses were conducted using Comprehensive Meta-Analysis Software (CMA) version 2 (Bio-stat, Englewood, NJ 2013). The analyses focused on calculating odds ratios (ORs) along with their corresponding 95% confidence intervals (CIs), providing a deeper understanding of the associations and probabilities. The variability of the included studies was assessed using I^2^ and Q statistics. If the I^2^ value is larger than 50% or if the Q value has a significance level of less than 0.05 or 0.10, it indicates the presence of heterogeneity [26]. A random-effects model was employed when heterogeneity was identified. In order to investigate potential factors that influence the link between intervention characteristics of technology-based interventions, subgroup analyses were performed to compare the magnitude of effects among specific groups. A p-value less than 0.05 was deemed to be statistically significant.

The assessment of publication bias was conducted through utilization of the Begg and Mazumdar rank correlation test, as well as the Duval and Tweedie’s trim and fill approach [27, 28]. The Begg’s adjusted rank correlation test yielded a p value of <0.05, indicating the presence of publication bias [27]. To ensure the robustness of our findings, identify any outliers, and maintain overall homogeneity (≤ 40%), we conducted a sensitivity analysis [26].

## Results

### Characteristics of included studies

**Fig 1:**
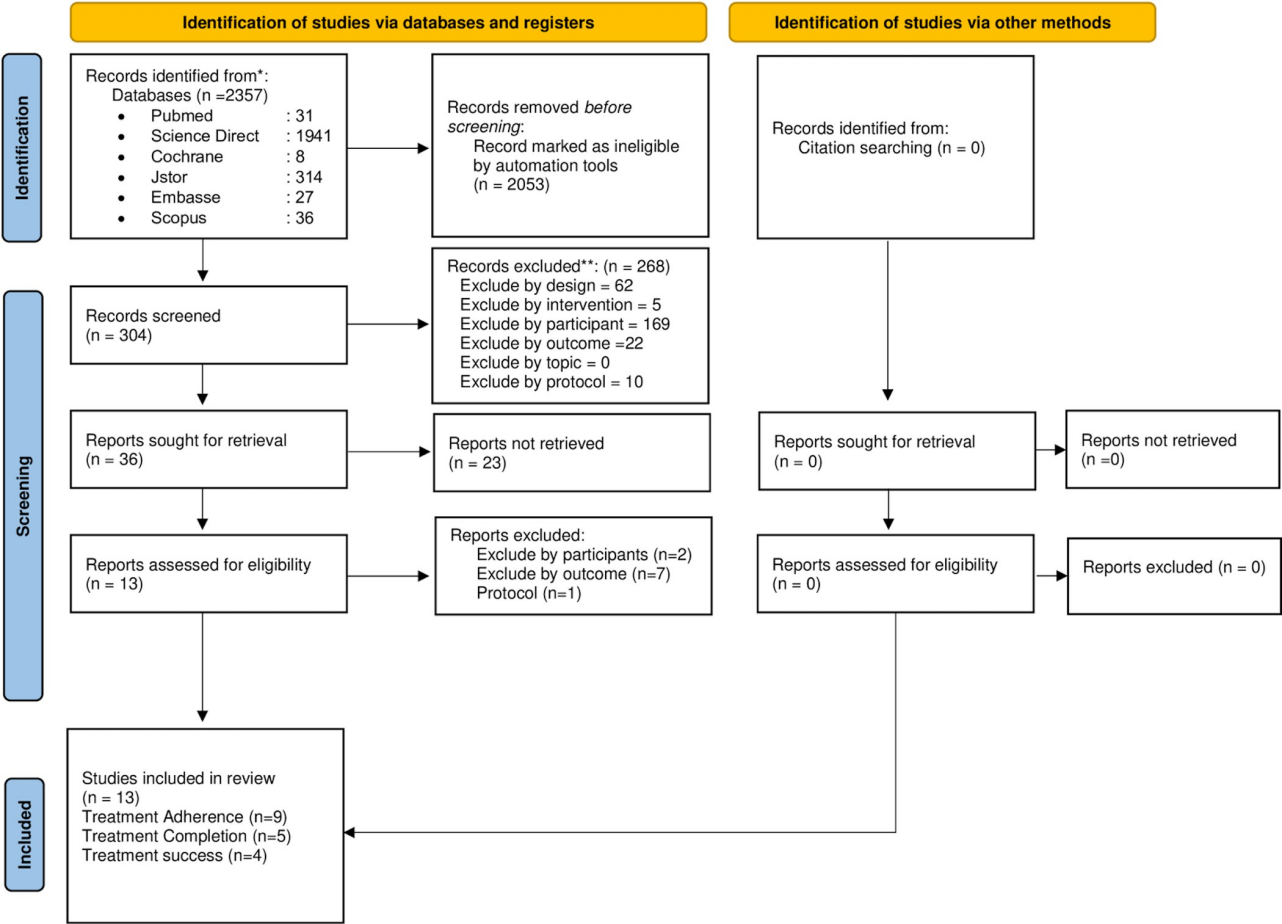
PRISMA.

The searches yielded a total of 2,357 citations. Among these, 2,053 records were marked as ineligible by automation tools. Additionally, 268 records were excluded due to unsuitability concerning study design, intervention, participants, outcomes, and protocol. Furthermore, 23 records were removed for ineligibility in reporting, and 10 records were excluded for unsuitability in terms of participants, outcomes, and protocol. No additional citations were identified through manual searching. Consequently, 13 randomized controlled trials (RCTs) were included in this review. The study selection process is summarized in Fig 1 using a PRISMA flowchart.

Characteristics of the included studies were summarized and shown in Table 1. Six articles including two different sets of data were extracted as two separate studies [1, 5, 17, 20, 26, 35]. Studies included in this review aimed to enhance treatment adherence, completion and overall success of tuberculosis patients. The studies can be categorized based on the type of intervention used. Text and phone-based interventions including SMS reminders, digital adherence technology, and two-way SMS communication were categorized into a text-based intervention group [5, 9, 20, 26] to analyze treatment adherence and success. Numerous studies have explored the effectiveness of video-based interventions in monitoring treatment adherence, completion, and success. Electronic Directly Observed Therapy (eDOT), Video Observed Therapy (VOT), and Video Directly Observed Therapy (VDOT) were classified under the VOT Intervention Group [8, 11, 17, 35, 36]. Moreover, the digital Medication Event Reminder and Monitor (MERM) system was evaluated to assess treatment adherence and success [16, 29]. Video Observed was utilized as a means to analyze treatment adherence, completion, and success [30].

**Table 1:** Characteristics included study.

Author, Year	Participants’ Characteristics	Intervention & Purpose	Interventions Characteristics	Provided by	Outcomes	Measurement tools
Acosta et al., 2022 [16]	Age: Mean (SD)	Purpose: To evaluate the effectiveness of a MERM on treatment success and treatment adherence	Intervention mode: Treatment monitor (dispenser pillbox), web-based and text-based	Health Professional trained	• Treatment success •Treatment adherence	• Self-reported •Self-reported
	I: 18–35 years: 41 (83.7) 36–59 years: 4 (8.2) >60 years: 4 (8.2)	I: MERM C: DOT	Frequency: 3 times a day			
	C: 18–35 years: 42 (79.2) 36–59 years: 8 (15.1) >60 years: 3 (5.7)		Duration: 4 months			
			Content: • Monitoring patients’ treatment •Possible connectivity problems •Reminding dose			
			Phase: treatment			
Belknap et al., 2018 [19]	Sample size:I: 315 C: 328	Purpose: To compare treatment completion and safety of once-weekly isoniazid and rifapentine by self-administration versus direct observation		-	Treatment completion	Self-reported
	Age (median) I: 38 (27–49)	I: SAT with reminder or once-weekly text message reminder. C: DOT	Intervention Mode: Text and phone-based for reminder			
			Frequency: Monthly follow-up visits			
			Duration: 4 months			
	C: 36 (27–48)		Content: Text massage reminder			
			Phase: treatment			
Bediang et al., 2018 [4]	Sample: 279 I: 137 C: 142	Purpose: evaluate the effectiveness of daily Short Message Service reminder in increasing treatment adherence	Intervention mode: Text-based and telephone based	Health Professional	• Treatment success •Treatment adherence •Patient cured rate	• Self-reported •Patient cured rate
	Age 18–25 year I: 22 (16.1%) C: 34 (23.9%)	I: SMS C: DOT	Frequency: Daily SMS Reminders			
	26–40 year I: 84 (61.3%) C: 79 (55.7%)		Duration: 6 months			
	41–55 year I: 26 (18.9%) C: 20 (14.1%)		Content: 1. SMS Reminders to take TB Treatment 2. Encouraging Messages every 2 weeks 3. Phone call			
	56–80 year I: 5 (93.7%) C: 9 (6.3%)		Phase: Treatment			
Browne et al., 2019 [30]	Sample size I: 41 C: 20	Purpose: To evaluate the effectiveness of wirelessly/video conference observed therapy (WOT) on daily adherence to medication.	Intervention mode: Web-based dashboard monitoring and support, text, and telephone	Health worker	Treatment adherence	Self-reported
	Age: Mean (SD) I: 41 (16) C: 45 (17)	I: WOT C: DOT	Content technology based: • Monitoring the ingestion of medication weekly •Following up using text massage and phone call within 24 hours if ingestion of Rifamate was not conformed			
			Frequency: Duration: 4 months			
			Phase: treatment			
Burzynski et al., 2022 [41]	Sample size I: 113 C: 103	Purpose: To examine the effectiveness of electronic DOT in increasing patient’s medication adherence	Intervention mode: Video conference and mobile Apps	TB clinic staff	Treatment completion	Self-reported
	Age Median (range): 41 (16–73) High education: 34 (79%)	I: Electronic DOT (Video conference) C: DOT	Frequency: 20 times			
			Duration: -			
			Content: Schedule and observation for medication Phase: treatment			
			Phase: treatment			
Cattamanchi et al., 2021 [21]	Sample size I: 987 C: 463	Purpose: To evaluate the effectiveness of digital adherence technology (Text based) for TB treatment	Intervention mode: Text and phone-based	Staff at health facilities	Treatment success	Uganda NTLP guidelines
	Age: Mean (SD) I: 38.9 (14.2) C: 39.2 (14.3)	I: Text based C: Usual Care	Frequency: -			
	Education: -		Duration: -			
			Content: • Daily automated messages dosing reminders •Daily phone call for Educational and motivational messages			
			Phase: Treatment			
Doltu et al., 2021 [42]	Sample: 169 I: 83 C: 86	Purpose Compared adherence and short and long-term between DOT and VOT group	Intervention mode: Asynchronous video observed therapy	TB Staff	Treatment adherence	History Treatment
	Average Age: 38.5 (46.8) High education: 27 (16%)	I: VOT C: DOT	Frequency: Pills swallowing times			
			Duration: 3 Months Content: Patients record their medication ingestion			
			Phase: Treatment			
Guo et al., 2019 [43]	Sample: 405 I: 203 C: 202	Purpose: Clinical and cost benefit of VDOT, compared with DOT service.	Intervention mode: Video-based	Staff Clinics	• Treatment adherence •Treatment complete •Patient cured rate	Self-reported
	Age: I: 40.2 (16.1) C: 44.3 (17.7)	I: VDOT C: DOT	Frequency: Pills swallowing times			
	High education: I: 170 (83.7%) C: 161 (79.7%)		Duration: -			
			Content: -			
			Phase: Treatment			
Johnston et al., 2017 [44]	Sample: 358 I: 170 C: 188	Purpose: to assess the effectiveness of two-way SMS communication on treatment completion	Intervention mode: Text-based	Clinic Nurser	• Treatment completion •Treatment adherence	Self-reported
	Median age: I: 45 (34–55) C: 42 (33–50)	I: Text message C: DOT	Frequency: Once weekly on Monday			
	High education: I: 145 (85%) C: 177 (94%)		Duration: 12 Months			
			Content: SMS Reminders to take TB Treatment			
			Phase: Treatment			
Louwagie, et al., 2022 [31]	Sample: 574 I: 283 C: 291	Purpose: To investigate the effectiveness ProLife on treatment success and medication adherence	Intervention mode: Counselling and text-based	TB Staff	• Treatment success •Treatment adherence	Self-reported
	Age: I: 38.56 (11.15) C: 39.37 (12.60)	I: ProLife (Text-based) C: Usual Care	Frequency: Counseling: 15–20 minutes 1 month apart Motivational message 2 times per week over 12 weeks Reminding messages			
			Duration: 12 weeks			
			Content: Information related to TB Augmenting motivation Reminding messages			
			Phase: Treatment			
	High education I: 8 (2.8%) C: 24 (8.2%)					
Manyazewal et al., 2022 [29]	Sample size: I: 57 C: 57	Purpose: To assess the effectiveness of digital medication event reminder and monitor (MERM) device-observed self-administered therapy in improving adherence and treatment outcomes	Intervention Mode: MERM	Health care	Treatment adherence	Self-reported
	Age: ≥ 18 years Mean (SD): 32.9 (11.07)	I: MERM C: DOT standard care	Freq: -			
			Duration: 15 days			
			Content: Participants received a 15-day tuberculosis medication supply in the evriMED500® MERM device to self-administer and return every 15 days			
			Phase: treatment			
Ravenscroft et al., 2020 [32]	Sample: 197 I: 98 C: 99	Purpose: the effectiveness of VOT compared to clinic-based DOT in improving medication adherence	Intervention mode: Video-based (Mobile-Apps)	Staff Clinics	• Treatment adherence •Treatment success	Self-reported
	Age: I: 38.73 (13.95) C: 38.28 (14.11)	I: VOT C: DOT	Frequency: Pills swallowing times			
			Duration: 3 Months			
			Content: Video recording procedure and detailed instructions of how to show that they swallowed their medication Report the side effect of medication			
			Phase: Treatment			
Story et al., 2019 [45]	Sampel Size: I: 112 C: 114	Purpose: To test the effectiveness of VOT for supporting treatment completion in patients with active tuberculosis in England.	Intervention Mode: Record and send videos of every dose ingested 7 days per week using smartphone app developed by researcher (Mobile-Apps)	Centralized service in London	Treatment completion	Semi-structured questionnaire
	Age: 16–54 years old I: 106 (93%)C: 99 (88%)	I: VOT C: DOT	Frequency: 7 days per week			
	≥55 years old I: 8 (7%) C: 13 (12%)		Duration: -			
			Content: • Trained treatment observers viewed the videos through a password-protected website • Patient are encouraged to report the side effect through the video			

SD, standar deviation; I, intervensi; C, control; DOT, directly observed therapy; VOT, video observed therapy; VDOT, video directly observed therapy; MERM, medication event reminder monitor system; SAT, self-administered therapy; SMS, short message service.

### The effectiveness of technology-based intervention on treatment adherence

Nine studies were included in the meta-analysis for treatment adherence. The technology-based interventions consisted of two MERM, three text-based, one video conference, and three VOT. The meta-analysis pooled effect estimate, represented by the odds ratio of 2.08 (95% CI: 1.70, 2.54), suggests a significant association between the variables for technology-based interventions. The assessment of heterogeneity indicated high heterogeneity among the included studies with I^2^: 90.8 (p-value: < .000).

**Fig 2:**
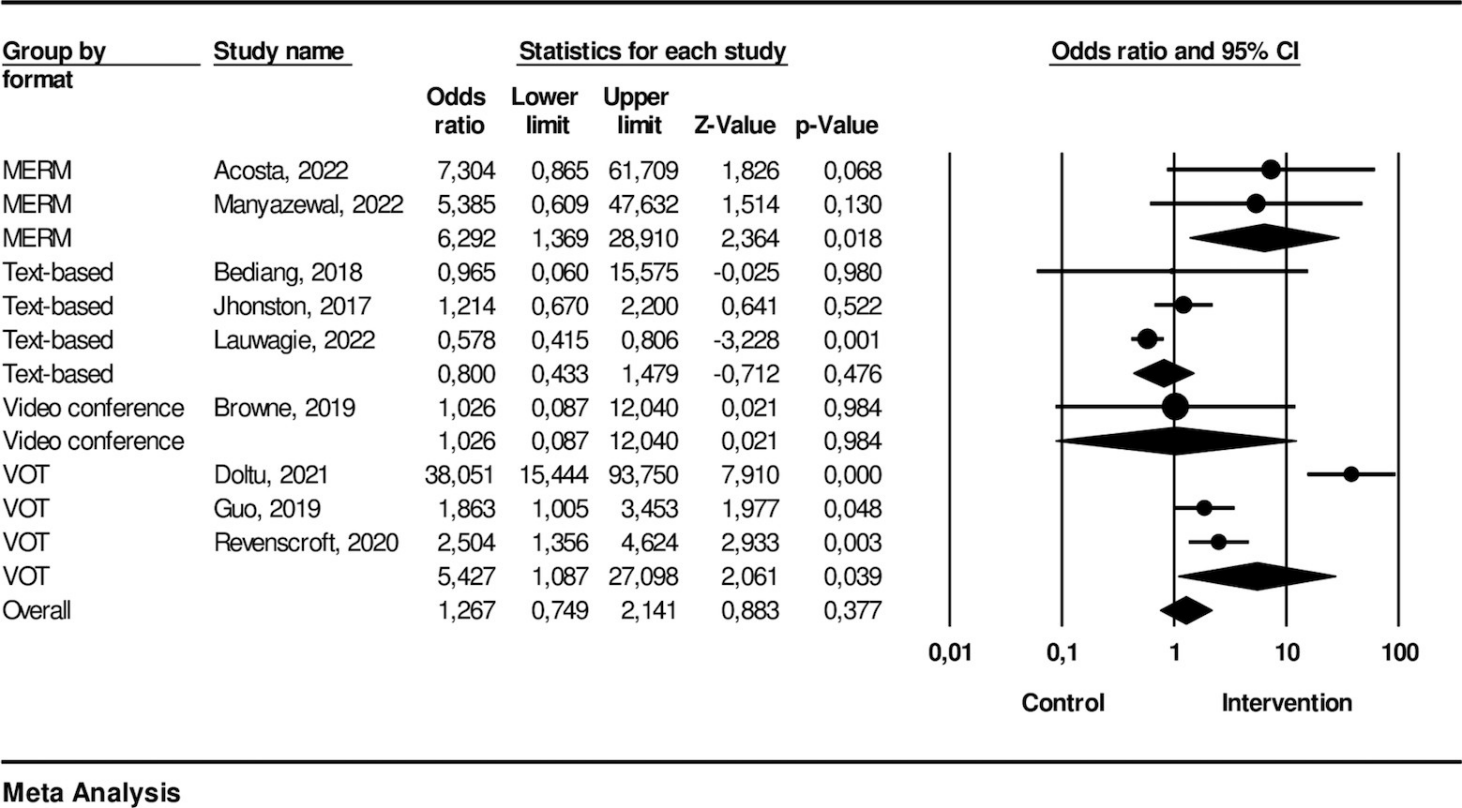
Treatment adherence.

From the subgroup analysis, we found that the type of intervention using MERM had a significant association with OR 6.29 (95% CI: 1.37, 28.9). The text-based intervention indicated no significance with OR 0.80 (95% CI: 0.43, 1.48). The video conference suggested a significant association with OR 1.03 (95% CI: 0.09, 12.4), and the VOT showed a significant association with OR 5.43 (95% CI:1.08, 27.1). The effectiveness of each technology-based intervention on treatment adherence is described in Fig 2.

### The effectiveness of technology-based intervention on treatment completion

Five studies were included in the meta-analysis assessing treatment completion. The interventions included two text-based, one video conference, and two VOT approaches, compared with DOT in the control group. The results indicated that participants who had technology-based interventions were more likely to complete the medication program compared to those in the control group (OR: 1.9; 95% CI: 1.46, 2.42; p-value: < .001) with I^2^: 82.26 (p-value: < .001) indicating high heterogeneity.

**Fig 3:**
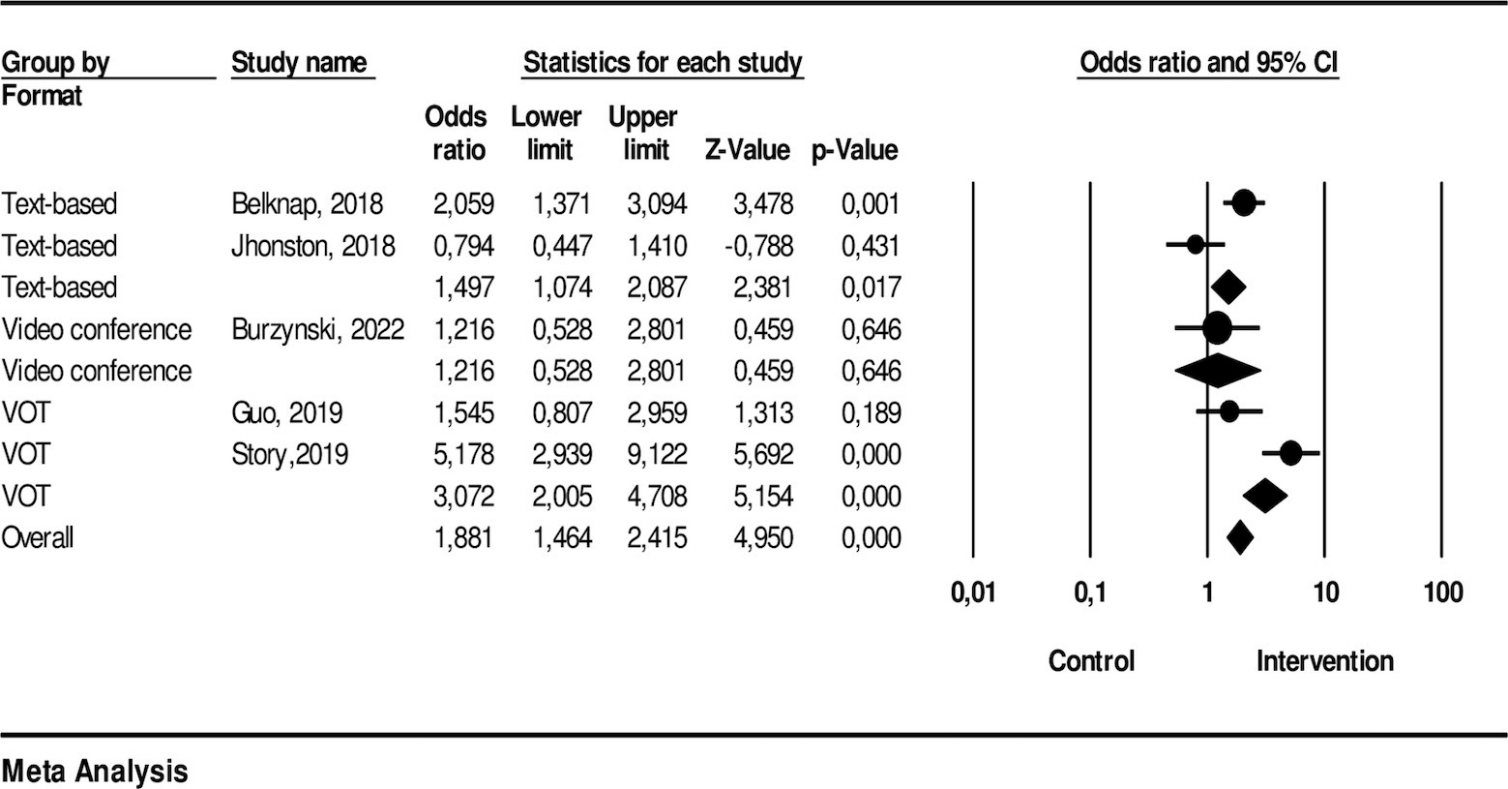
Completion of medication.

From the analysis, we found that the text-based interventions demonstrated a significant effect, showing an odds ratio (OR) of 1.50 (95% CI: 1.07, 2.09). The video conference interventions, on the other hand, did not show a statistically significant effect with an OR of 1.22 (95% CI: 0.53, 2.80). The Voice-over-Internet Protocol (VOT) interventions exhibited a significant effect with an OR of 3.07 (95% CI: 2.01, 4.71). Fig 3 depicts the effectiveness of each technology-based interventions delivery method on completion of medication.

### The effectiveness of technology-based intervention on treatment success

This meta-analysis assessed treatment success, encompassing data from five studies that employed different intervention approaches including one utilizing MERM, three employing text-based methods, and one utilizing video conference technology. The results indicated that participants who had technology-based interventions were more likely to be successful in treatment compared to those in the control group (OR: 1.66; 95% CI: 1.35–2.05; p-value: < .001) with an I^2^ of 84.02 (p-value: < .001) indicating high heterogeneity.

**Fig 4:**
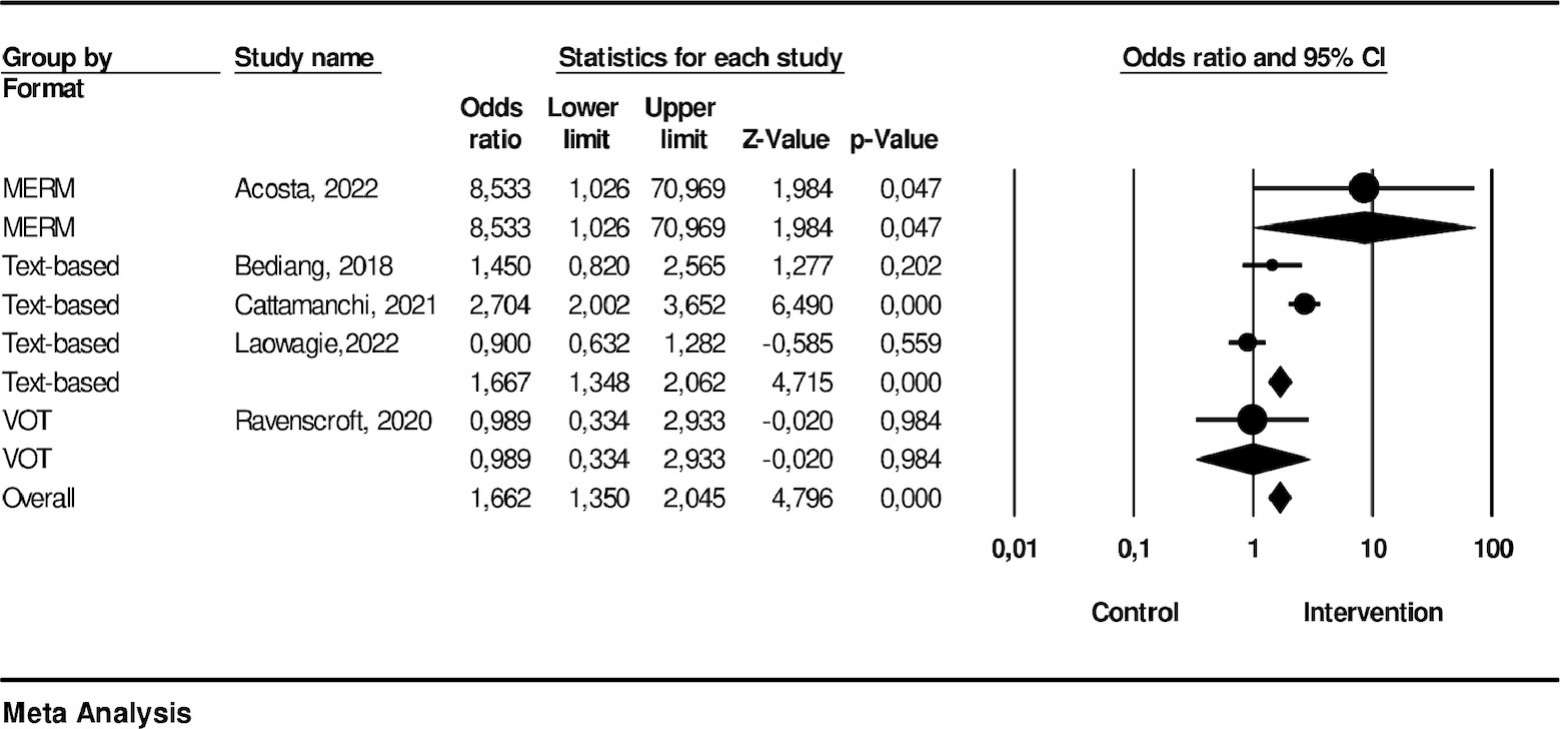
Treatment success.

Sub-group analysis revealed that the MERM (Mobile-Enhanced Remote Monitoring) interventions, represented by the study conducted by Acosta [16], revealed a significant effect with an odds ratio (OR) of 8.53 (95% CI: 1.03, 70.97). The text-based interventions, encompassing studies by Bediang [4], Cattamanchi [21], and Louwagie [31], showed a significant effect on treatment success (OR: 1.66; 95% CI: 1.35–2.06; p-value: < .001). Moreover, the VOT interventions, as represented by Ravenscroft et al. [32], did not exhibit a significant effect (OR = 1.66, 95% CI: 1.35, 2.05). The effectiveness of each intervention’s platform is shown in Fig 4.

### Risk of bias of included trials

**Fig 5:**
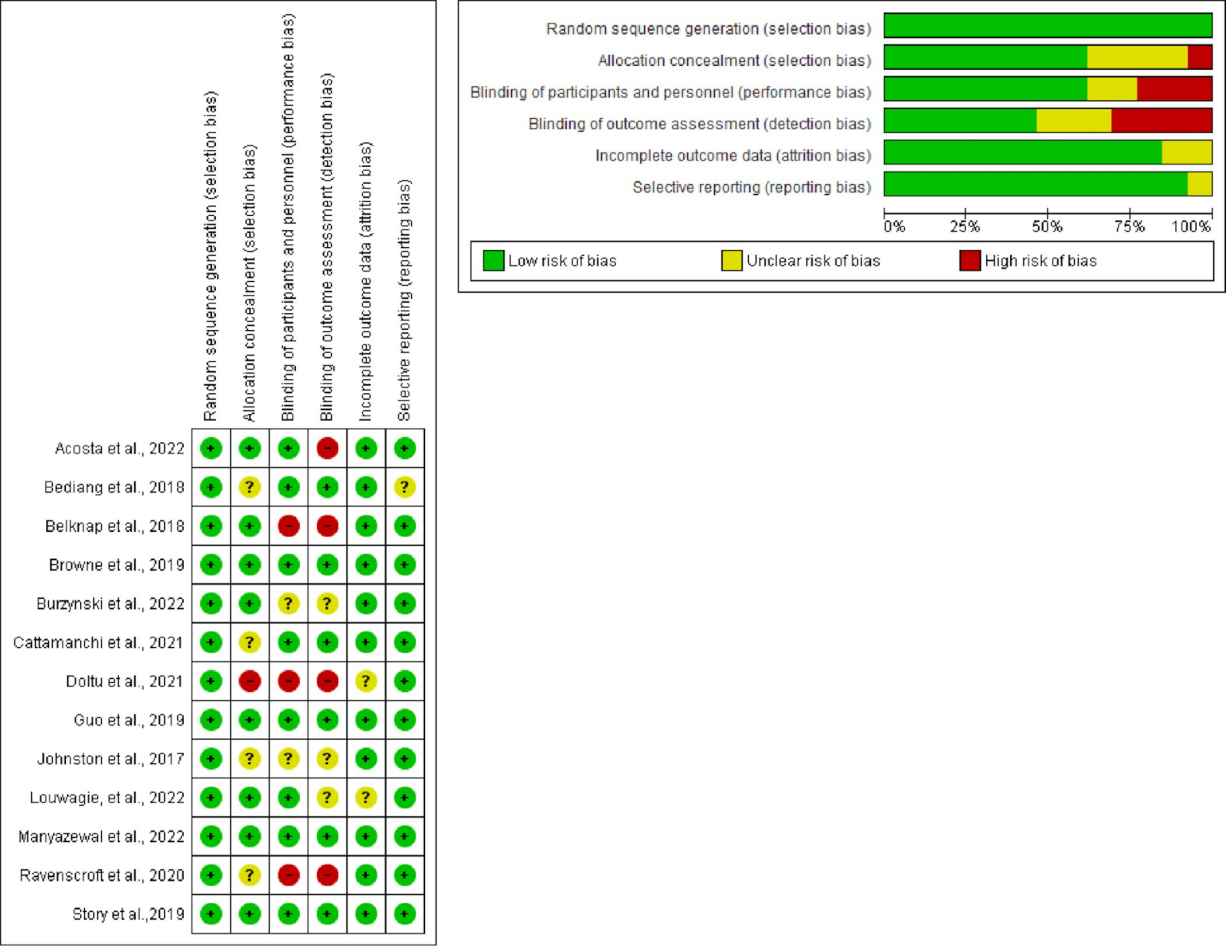
ROB.

Thirteen trials reported sufficient random sequence generation, while only eight trials reported allocation concealment. Although some studies explained the challenges of blinding in these interventions, eight trials initiated either single or double blinding. However, only six trials clearly stated the blinding of outcome assessors. In total, eleven trials demonstrated a low risk of incomplete outcome data, whereas twelve trials exhibited a low risk of selective outcome reporting. Fig 5 present the risk of bias judgments.

### Publication bias

In order to evaluate the presence of publication bias, we employed Begg and Mazumdar’s rank correlation test, utilizing Kendall’s tau statistics with a continuity correction. The Kendall’s tau statistic for treatment adherence was determined to be 0.3, with a corresponding z-score of 0.73 and a p-value of 0.46. The results of our analysis showed that the Kendall’s tau statistics for treatment completion and treatment success were 0.17, z = 0.62, p = 0.53 and 0.00, z = 0.00, p = 1.00, respectively. These findings indicate that there was no publication bias.

### Sensitivity analysis

No outliers were identified in the sensitivity analysis when the study with the greatest effect size on treatment adherence, treatment completion and treatment success was excluded. Significant ORs were obtained for treatment adherence (OR: 1.56; 95% CI: 0.82, 2.95), treatment completion (OR: 1.61; 95% CI: 0.85, 3.06), and treatment success (OR: 1.42; 95% CI: 0.74, − 2.74), and these values suggest that the findings were robust.

## Discussion

This study represents the first meta-analysis of randomized controlled trials (RCTs) that examines the efficacy of various treatment methods, including Directly Observed Therapy (DOT), on tuberculosis (TB) patient outcomes. Our analysis has demonstrated that tailored technology-based interventions are crucial for achieving better treatment adherence, completion, and success rates, underscoring the importance of utilizing unique technology-based approaches to enhance TB management. Prior research has indicated that digital adherence technologies (DATs), such as feature phone-based and smartphone-based technologies, digital pillboxes, and ingestible sensors, can enable more patient-centric approaches for monitoring TB medication adherence than current DOT models [15]. A randomized trial conducted in Kenya has shown a reduction in poor outcomes (on-treatment death, loss to follow-up, or treatment failure), primarily by reducing loss to follow-up, when using SMS reminders and an unstructured supplementary service data intervention [33]. An individually randomized trial’s per-protocol analysis has demonstrated an increase in treatment success (cured or completed treatment) among patients who received a real-time medication event reminder monitor. Patients received SMS reminders if the monitor was not opened at the scheduled treatment time, escalating to sending an SMS to a previously designated relative or treatment supporter if the monitor remained unopened.

The present study aimed to conduct a comprehensive analysis of technology-based interventions for tuberculosis (TB) patients and to compare their efficacy with the traditional Directly Observed Therapy (DOT) in terms of adherence, completion, and success rates. The study revealed that personalized strategies play a pivotal role in enhancing TB treatment as different technological interventions produced varied outcomes. The findings indicated that technology-based interventions are associated with improved treatment adherence, consistent with the research conducted by Liu et al. [8]. It is noteworthy that the Mobile-Enhanced Remote Monitoring (MERM) intervention had the most significant impact, owing to its employment of mobile apps for real-time medication monitoring. This approach empowered both patients and healthcare providers, leading to higher adherence rates and better overall treatment outcomes for TB management [7]. Additionally, interventions like video conference and Video Observed Therapy (VOT) also showed significant associations, underscoring the potential of technology-based solutions to enhance treatment adherence. Prior research has indicated that Video Observed Therapy (VOT) significantly improves medication adherence compared to the conventional Directly Observed Therapy (DOT). A randomized controlled trial (RCT) study reported that participants in the intervention arm exhibited substantially better compliance than those who received family-based DOT. These findings suggest that VOT can be a promising alternative to DOT for promoting medication adherence, especially in settings where family support is not readily available. Further research is needed to explore the long-term effectiveness and cost-effectiveness of VOT in diverse patient populations and healthcare settings [34]. Given the significant potential of technology to enhance medication adherence, it is highly recommended to consider utilizing VOT in settings where TB patients are prevalent in the productive age group, mobility is high, smartphone usage is common, and internet coverage is comprehensive [15].

In the sphere of evaluating the completion of treatments, interventions in the form of text messages have demonstrated their utility. This observation is consistent with prior research, which has established that reminder applications and technologically-advanced pillboxes have led to significant improvements in treatment outcomes in contrast to conventional care [35]. Conversely, video conferences did not demonstrate a meaningful impact, whereas Video Observed Therapy (VOT) interventions yielded a favourable outcome in a comparative study analysing treatment completion rates, it was determined that the use of Video Observed Therapy (VOT) did not significantly improve completion rates when compared to Directly Observed Therapy (DOT). These findings are consistent with prior research on the matter. While the results of the study suggest that VOT may not be superior to DOT in terms of treatment completion [36]. Nevertheless, the overall analysis did not yield a statistically significant distinction, underscoring the necessity for more investigation and honing in on comprehending the influence of various intervention models.

According to the study, previous research has shown that MERM interventions may have a positive impact on treatment success. This is consistent with findings on the effectiveness of reminder apps and smart pillboxes in a programmatic context [35]. Conversely, VOT interventions displayed a notable correlation with favourable outcomes and played a key role in the overall triumph of technology-based interventions in enhancing treatment results. According to a previous study, patients receiving virtual directly observed therapy (vDOT) had the same rate of successful treatment outcomes as those receiving in-person DOT. The completion/cure rate for vDOT was 96%, with only 2% of patients being transferred to a different program and 2% passing away. In comparison, the completion/cure rate for in-person DOT was slightly lower, at 90%, with 5% of patients being transferred to another program and 4% passing away. However, the difference in success rates between the two groups was not statistically significant. Furthermore, patients in both groups experienced similar microbiological outcomes, taking an average of 48 days to culture conversion. These findings suggest that technology-based interventions, such as vDOT, have potential to improve treatment outcomes [37].

Our research has shed light on the evolving approach to managing tuberculosis through the incorporation of technology. This opens up exciting prospects for tailored interventions that have the potential to significantly enhance adherence, completion, and outcomes of treatment. Reminder message content has been proven to play a crucial role in determining patient adherence according to previous studies [38]. It is important to note that adopting a patient-centred approach can significantly enhance adherence, completion, and treatment outcomes. This is based on the well-established theory that intrinsically motivated individuals are more inclined to engage and persist in tasks [39, 40]. It is imperative for further studies to delve into the underlying mechanisms of these effects and concentrate on refining and customizing technology-driven interventions to optimize benefits for tuberculosis patients worldwide.

### Limitation

Although the study offers valuable insights, it is crucial to acknowledge its limitations. First, the level of heterogeneity among included studies was high. Nevertheless, we conducted a subgroup analysis to investigate the variability within the data. Second, the individual studies shown an elevated susceptibility to bias resulting from allocation concealment, blinding of participants, and blinding of the outcome. Hence, forthcoming randomized controlled trials (RCTs) may necessitate the inclusion of comprehensive details pertaining to the allocation concealment and blinding of participants as well as the outcome.

## Conclusions

The insightful findings from our meta-analyses shed light on the effectiveness of technology-based interventions, categorized into Mobile-Enhanced Remote Monitoring (MERM), text-based, video conference, and Video Observed Therapy (VOT). These analyses reveal significant associations with treatment adherence and completion, highlighting the immense potential of technology to enhance patient outcomes in tuberculosis management. However, the observed heterogeneity among the studies emphasizes the need for a nuanced and tailored approach, considering different intervention types and potential sources of variation to achieve optimal effectiveness.

## Supporting information

S1 Table Search strategy. (DOCX)

S2 Table List of the included studies for final analysis. (DOCX)

S3 Table List of the excluded studies. (DOCX)

S4 Table Raw data. (DOCX)

S5 Table Risk of bias among included studies. (DOCX)

S6 Table PRISMA checklist. (DOCX)
