# Supplementary material for: A meta-analysis of technology-based interventions on treatment adherence and treatment success among TBC patients
Source: PLoS One. 2024 Dec 2;19(12):e0312001. doi: 10.1371/journal.pone.0312001 (PMC11611106; doi:10.1371/journal.pone.0312001)
Supplement: S1 Table — (DOCX) [file pone.0312001.s001.docx]

**S1 Table. Search strategy**

| **Databases** | **Keywords** |
| --- | --- |
| PubMed | ((Tuberculosis[Title/Abstract] OR pulmonary tuberculosis[Title/Abstract] OR sputum-positive tuberculosis[Title/Abstract] AND ((y_5[Filter]) AND (ffrft[Filter]) AND (randomizedcontrolledtrial[Filter]) AND (english[Filter]))) AND (adherence[Title/Abstract] OR compliance[Title/Abstract] OR concordance[Title/Abstract] OR treatment[Title/Abstract] OR anti-tuberculosis medication[Title/Abstract] OR intervention[Title/Abstract] OR therapy[Title/Abstract] OR DOT[Title/Abstract] OR DOTS[Title/Abstract] OR Screening[Title/Abstract] OR treatment completion[Title/Abstract] OR completion rate[Title/Abstract] OR cure rate[Title/Abstract] AND ((y_5[Filter]) AND (ffrft[Filter]) AND (randomizedcontrolledtrial[Filter]) AND (english[Filter])))) AND (health education[Title/Abstract] OR technology based intervention[Title/Abstract] OR Telephone[Title/Abstract] OR cellular phone[Title/Abstract] OR wireless technology[Title/Abstract] OR reminder system[Title/Abstract] OR text[Title/Abstract] OR message[Title/Abstract] OR Phone text[Title/Abstract] OR mobile application[Title/Abstract] OR voice call[Title/Abstract] OR MMS[Title/Abstract] OR digital[Title/Abstract] OR website[Title/Abstract] OR m-Health[Title/Abstract] OR Mobile Health[Title/Abstract] OR tele-Counseling[Title/Abstract] OR teleconference[Title/Abstract] OR Video[Title/Abstract] OR educational technology[Title/Abstract] OR Instructional Technology[Title/Abstract] AND ((y_5[Filter]) AND (ffrft[Filter]) AND (randomizedcontrolledtrial[Filter]) AND (english[Filter]))) |
| ScienceDirect | Tuberculosis AND Adherence AND Technology based AND Randomized Controlled Trial |
| Cochrane | "tuberculosis" AND "patient compliance" AND "technologically" AND "randomized controlled trial" |
| JSTOR | Tuberculosis AND Adherence AND Technology based AND Randomized Controlled Trial |
| Embase | 'tuberculosis'/exp AND technology AND ('patient compliance'/exp OR 'adherence to therapy' OR 'adherence to treatment' OR 'compliance to therapy' OR 'compliance to treatment' OR 'patient adherence' OR 'patient compliance' OR 'patients` adherence' OR 'therapy adherence' OR 'therapy compliance' OR 'treatment adherence' OR 'treatment adherence and compliance' OR 'treatment compliance') AND 'randomized controlled trial'/exp |
| Scopus | TITLE-ABS-KEY(Tuberculosis)  AND TITLE-ABS-KEY(adherence)  AND TITLE-ABS-KEY(Technology) AND TITLE-ABS-KEY(Randomized Controlled Trial) |
