# Supplementary material for: A meta-analysis of technology-based interventions on treatment adherence and treatment success among TBC patients
Source: PLoS One. 2024 Dec 2;19(12):e0312001. doi: 10.1371/journal.pone.0312001 (PMC11611106; doi:10.1371/journal.pone.0312001)
Supplement: S2 Table — (DOCX) [file pone.0312001.s002.docx]

**S2 Table. List of the included studies for final analysis**

| **Included studies** |
| --- |
| Acosta et al., 2022^1^ |
| Bediang et al., 2018^2^ |
| Belknap et al., 2018^3^ |
| Browne et al., 2019^4^ |
| Burzynski et al., 2022^5^ |
| Cattamanchi et al., 2021^6^ |
| Doltu et al., 2021^7^ |
| Guo et al., 2019^8^ |
| Johnston et al., 2017^9^ |
| Louwagie, et al., 2022^10^ |
| Manyazewal et al., 2022^11^ |
| Ravenscroft et al., 2020^12^ |
| Story et al., 2019^13^ |

**References**

1. Acosta J, Flores P, Alarcón M, Grande-Ortiz M, Moreno-Exebio L, Puyen ZM. A

randomised controlled trial to evaluate a medication monitoring system for TB treatment. *Int J Tuberc Lung Dis*. 2022;26 (1):44–9. <https://doi.org/10.5588/ijtld.21.0373>.

1. Bediang G, Stoll B, Elia N, Abena JL, Geissbuhler A. SMS reminders to improve adherence and cure of tuberculosis patients in Cameroon (TB-SMS Cameroon): a randomised controlled trial. *BMC Public Health*. 2018;18(1):583. <https://doi.org/10.1186/s12889-018-5502-x>
2. Belknap R, Holland D, Feng PJ, Millet JP, Caylà JA, Martinson NA, et al. Self-administered versus directly observed once-weekly isoniazid and rifapentine treatment of latent tuberculosis infection: a randomized trial. *Ann Intern Med.* 2017;167(10):689–97. <https://doi.org/10.7326/M17-1150>.
3. Browne SH, Umlauf A, Tucker AJ, Low J, Moser K, Gonzalez Garcia J, Peloquin CA, Blaschke T, Vaida F, Benson CA. Wirelessly observed therapy compared to directly observed therapy to confirm and support tuberculosis treatment adherence: A randomized controlled trial. *PLoS Med*. 2019;16(10):e1002891. <https://doi.org/10.1371/journal.pmed.1002891>
4. Burzynski, J., Mangan, J. M., Lam, C. K., Macaraig, M., Salerno, M. M., deCastro, B. R., Goswami, N. D., Lin, C. Y., Schluger, N. W., Vernon, A., & eDOT Study Team. (2022). In-Person vs Electronic Directly Observed Therapy for Tuberculosis Treatment Adherence: A Randomized Noninferiority Trial. *JAMA Network Open*, 5(1), e2144210. <https://doi.org/10.1001/jamanetworkopen.2021.44210>
5. Cattamanchi A, Crowder R, Kityamuwesi A, Kiwanuka N, Lamunu M, Namale C, Tinka LK, Nakate AS, Ggita J, Turimumahoro P, Babirye D, Oyuku D, Berger C, Tucker A, Patel D, Sammann A, Turyahabwe S, Dowdy D, Katamba A. Digital adherence technology for tuberculosis treatment supervision: A stepped-wedge cluster-randomized trial in Uganda. *PLoS Med*. 2021;18(5):e1003628. <https://doi.org/10.1371/journal.pmed.1003628>
6. Doltu S, Ciobanu A, Sereda Y, Persian R, Ravenscroft L, Kasyan L, Truzyan N, Dadu A, Reid A. Short and long-term outcomes of video observed treatment in tuberculosis patients, the Republic of Moldova. *J Infect Dev Ctries*. 2021;15(9.1):17S–24S. <https://doi.org/10.3855/jidc.14601>.
7. Guo P, Qiao W, Sun Y, Liu F, Wang C. Telemedicine technologies and tuberculosis management: A randomized controlled trial. *Telemed J E Health*. 2020;26(9):1150–6. <https://doi.org/10.1089/tmj.2019.0190>.
8. Johnston JC, van der Kop ML, Smillie K, Ogilvie G, Marra F, Sadatsafavi M, Romanowski K, Budd MA, Hajek J, Cook V, Lester RT. The effect of text messaging on latent tuberculosis treatment adherence: a randomised controlled trial. *Eur Respir J*. 2018;51(2):1701488. <https://doi.org/10.1183/13993003.01488-2017>
9. Louwagie G, Kanaan M, Morojele NK, Van Zyl A, Moriarty AS, Li J, Siddiqi K, Turner A, Mdege ND, Omole OB, Tumbo J, Bachmann M, Parrott S, Ayo-Yusuf OA. Effect of a brief motivational interview and text message intervention targeting tobacco smoking, alcohol use and medication adherence to improve tuberculosis treatment outcomes in adult patients with tuberculosis: a multicentre, randomised controlled trial of the ProLife programme in South Africa. *BMJ Open*. 2022;12(2):e056496. <https://doi.org/10.1136/bmjopen-2021-056496>
10. Manyazewal T, Woldeamanuel Y, Holland DP, Fekadu A, Marconi VC. Effectiveness of a digital medication event reminder and monitor device for patients with tuberculosis (SELFTB): a multicenter randomized controlled trial. *BMC Med*. 2022;20(1):310. <https://doi.org/10.1186/s12916-022-02521-y>
11. Ravenscroft L, Kettle S, Persian R, Ruda S, Severin L, Doltu S, Schenck B, Loewenstein G. Video-observed therapy and medication adherence for tuberculosis patients: randomised controlled trial in Moldova. *Eur Respir J*. 2020;56(2):2000493. <https://doi.org/10.1183/13993003.00493-2020>.
12. Story A, Aldridge RW, Smith CM, Garber E, Hall J, Ferenando G, Possas L, Hemming S, Wurie F, Luchenski S, Abubakar I, McHugh TD, White PJ, Watson JM, Lipman M, Garfein R, Hayward AC. Smartphone-enabled video-observed versus directly observed treatment for tuberculosis: a multicentre, analyst-blinded, randomised, controlled superiority trial. *Lancet*. 2019;393(10177):1216–24. <https://doi.org/10.1016/S0140-6736(18)32993-3>
