# Supplementary material for: A meta-analysis of technology-based interventions on treatment adherence and treatment success among TBC patients
Source: PLoS One. 2024 Dec 2;19(12):e0312001. doi: 10.1371/journal.pone.0312001 (PMC11611106; doi:10.1371/journal.pone.0312001)
Supplement: S3 Table — (DOCX) [file pone.0312001.s003.docx]

**S3 Table. List of the excluded studies after a full-text review**

| **Author, year** | **Reasons of exclusion** |
| --- | --- |
| Bao et al., 2022^1^ | Did not report the outcome of interest |
| Byonanebye et al., 2021^2^ | Did not report the outcome of interest |
| Fernandes et al., 2022^3^ | Did not report the outcome of interest |
| Gashu et al., 2019^4^ | Did not report the outcome of interest |
| Majella et al., 2021^5^ | Did not report the outcome of interest |
| Manyazewal et al., 2022^6^ | Did not report the outcome of interest |
| Mwansa-Kambafwile et al., 2022^7^ | Did not report the outcome of interest |
| Sequeira-Aymar^8^ | Did not report the outcome of interest |
| Thompson et al., 2022^9^ | Did not report the outcome of interest |
| Wagstaff A et al., 2019^10^ | Did not report the outcome of interest |

**References**

1. Bao Y, Wang C, Xu H, Lai Y, Yan Y, Ma Y, Yu T, Wu Y. Effects of an mHealth Intervention for Pulmonary Tuberculosis Self-management Based on the Integrated Theory of Health Behavior Change: Randomized Controlled Trial. *JMIR Public Health Surveill*. 2022;8(7):e34277. <https://doi.org/10.2196/34277>
2. Byonanebye, D. M., Mackline, H., Sekaggya-Wiltshire, C., Kiragga, A. N., Lamorde, M., Oseku, E., King, R., & Parkes-Ratanshi, R. (2021). Impact of a mobile phone-based interactive voice response software on tuberculosis treatment outcomes in Uganda (CFL-TB): a protocol for a randomized controlled trial. *Trials*, 22(1), 391. <https://doi.org/10.1186/s13063-021-05352-z>
3. Fernandes L, Narvekar A, Lawande D. Efficacy of smoking cessation intervention delivered through mobile tele-counseling among smokers with tuberculosis in a Revised National Tuberculosis Control Program. *Indian J Tuberc.* 2022;69(2):207–12. <https://doi.org/10.1016/j.ijtb.2021.08.017>
4. Gashu KD, Gelaye KA, Lester R, Tilahun B. Combined effect of pill refilling and self-medication reminder system on patients' adherence to tuberculosis treatment during continuation phase in Northwest Ethiopia: a study protocol for randomised controlled trial. *BMJ Health Care Inform*. 2019;26(1):e100050. <https://doi.org/10.1136/bmjhci-2019-100050>
5. Majella MG, Thekkur P, Kumar AM, Chinnakali P, Saka VK, Roy G. Effect of mobile voice calls on treatment initiation among patients diagnosed with tuberculosis in a tertiary care hospital of Puducherry: a randomized controlled trial. *J Postgrad Med*. 2021;67(4):205–12. <https://doi.org/10.4103/jpgm.JPGM_1105_20>
6. Manyazewal T, Woldeamanuel Y, Fekadu A, Holland DP, Marconi VC. Effect of Digital Medication Event Reminder and Monitor-Observed Therapy vs Standard Directly Observed Therapy on Health-Related Quality of Life and Catastrophic Costs in Patients With Tuberculosis: A Secondary Analysis of a Randomized Clinical Trial. *JAMA Netw Open*. 2022;5(9):e2230509. <https://doi.org/10.1001/jamanetworkopen.2022.30509>
7. Mwansa-Kambafwile JRM, Chasela C, Levin J, Ismail N, Menezes C. Treatment initiation among tuberculosis patients: the role of short message service (SMS) technology and ward-based outreach teams (WBOTs). BMC *Public Health*. 2022;22(1):318. <https://doi.org/10.1186/s12889-022-12736-6>
8. [Sequeira-Aymar E, Cruz A, Serra-Burriel M, di Lollo X, Gonçalves AQ, Camps-Vilà L, Monclus-Gonzalez MM, Revuelta-Muñoz EM, Busquet-Solé N, Sarriegui-Domínguez S, Casellas A, Llorca MRD, Aguilar-Martín C, Jacques-Aviñó C, Hargreaves S, Requena-Mendez A, CRIBMI (IS-MiHealth) Working Group. Improving the detection of infectious diseases in at-risk migrants with an innovative integrated multi-infection screening digital decision support tool (IS-MiHealth) in primary care: a pilot cluster-randomized-controlled trial. *J Travel Med*. 2022;29(7):taab100. <https://doi.org/10.1093/jtm/taab100>
9. Thompson RR, Kityamuwesi A, Kuan A, Oyuku D, Tucker A, Ferguson O, Kunihira Tinka L, Crowder R, Turyahabwe S, Cattamanchi A, Dowdy DW, Katamba A, Sohn H. Cost and cost-effectiveness of a digital adherence technology for tuberculosis treatment support in Uganda. *Value Health*. 2022;25(6):924–30. <https://doi.org/10.1016/j.jval.2021.12.002>
10. Wagstaff A, van Doorslaer E, Burger R. SMS nudges as a tool to reduce tuberculosis treatment delay and pretreatment loss to follow-up: A randomized controlled trial. *PLoS One*. 2019;14(6):e0218527. <https://doi.org/10.1371/journal.pone.0218527>
