# Supplementary material for: A meta-analysis of technology-based interventions on treatment adherence and treatment success among TBC patients
Source: PLoS One. 2024 Dec 2;19(12):e0312001. doi: 10.1371/journal.pone.0312001 (PMC11611106; doi:10.1371/journal.pone.0312001)
Supplement: S4 Table — (DOCX) [file pone.0312001.s004.docx]

**S4 Table. Effectiveness of technology-based intervention on treatment**

**S4.1. Treatment adherence**

| **Study Citation** | **Data Extractors** | **Date of Data Extraction** | **Session**  **(Reason for inclusion)** | **Extracted Data** | | | | |
| --- | --- | --- | --- | --- | --- | --- | --- | --- |
|  |  |  |  | **Intervention** | | **Control** | | **p-value** |
|  |  |  |  | **n** | **N** | **n** | **N** |  |
| Acosta et al., 2022 | Huda, MH | 17 November 2022 | Increase adherence (treatment support) | 48 | 49 | 45 | 53 | 0,0322 |
| Bediang et al.,2018 | Huda, MH | 17 November 2022 | Increase adherence (treatment support) | 111 | 137 | 106 | 142 | 0.20 |
| Cattamanchi et al.,2021 | Huda, MH | 17 November 2022 | Adherence and completion of TB treatment (treatment Support) | 401 | 463 | 696 | 987 | 0.001 |
| Laouwagie et al.,2022 | Huda, MH | 17 November 2022 | Increase adherence (treatment support) | 192 | 283 | 204 | 291 | - |
| Ravenscroft L et al.,2020 | Huda, MH | 17 November 2022 | Increase adherence (treatment support) | 91 | 98 | 92 | 99 | - |

N, sample size; n, number of participant followed treatment.

**S4.2.Treatment adherence**

| **Study Citation** | **Data Extractors** | **Date of Data Extraction** | **Session**  **(Reason for inclusion)** | **Extracted Data** | | | |
| --- | --- | --- | --- | --- | --- | --- | --- |
|  |  |  |  | **Intervention** | | **Control** | |
|  |  |  |  | **n** | **N** | **n** | **N** |
| Acosta et al., 2022 | Huda, MH | 17 November 2022 | Increase adherence (treatment support) | 48 | 49 | 46 | 53 |
| Bediang et al.,2018 | Huda, MH | 17 November 2022 | Increase adherence (treatment support) | 136 | 137 | 141 | 142 |
| Browne et al.,2019 | Rahman, MF | 30 November 2022 | Increase adherence (treatment support) | 39 | 41 | 19 | 20 |
| Doltu et al.,2021 | Rahman, MF | 30 November 2022 | Avot treatment  (treatment Support) | 75 | 83 | 17 | 86 |
| Guo et al., 2019 | Rahman, MF | 30 November 2022 | Increase adherence (treatment support) | 185 | 203 | 171 | 202 |
| Johnston et al., 2017 | Rahman, MF | 30 November 2022 | Increase adherence (treatment support) | 131 | 153 | 152 | 183 |
| Lauwagie et al., 2022 | Rahman, MF | 30 November 2022 | Increase adherence (treatment support) | 138 | 283 | 181 | 291 |
| Manyazewal, 2022 | Rahman, MF | 30 November 2022 | Self administred  (treatment support) | 57 | 100 | 55 | 57 |
| Ravenscroft L et al.,2020 | Huda, MH | 17 November 2022 | Increase adherence (treatment support) | 75 | 98 | 56 | 99 |

N, sample size; n, number of participant followed treatment.

**S4.3 Treatment completion of medication**

| **Study Citation** | **Data Extractors** | **Date of Data Extraction** | **Session**  **(Reason for inclusion)** | **Extracted Data** | | | | | |
| --- | --- | --- | --- | --- | --- | --- | --- | --- | --- |
|  |  |  |  | **Intervention** | | **Control** | | **p-value** | **Mean diff** |
|  |  |  |  | **n** | **N** | **n** | **N** |  |  |
| Belknap et al.,2018 | Rahman, MF | 30 November 2022 | Self Administred  (treatment Support) | 241 | 315 | 286 | 328 | - | - |
| Burzynski et al., 2022 | Rahman, MF | 30 November 2022 | Monitoring Tuberculosis treatment (treatment Support) | 101 | 113 | 90 | 103 | - | -4,9 |
| Guo et al., 2019 | Rahman, MF | 30 November 2022 | increase adherence (treatment support) | 186 | 203 | 177 | 202 | 0,12 | - |
| Johnston et al., 2018 | Rahman, MF | 30 November 2022 | increase adherence (treatment support) | 131 | 170 | 152 | 188 | - | - |
| Story et al.,2019 | Rahman, MF | 30 November 2022 | Monitoring Tuberculosis treatment (treatment Support) | 112 | 112 | 35 | 114 | - | - |

N, sample size; n, number of participant followed treatment.
