# Supplementary material for: A meta-analysis of technology-based interventions on treatment adherence and treatment success among TBC patients
Source: PLoS One. 2024 Dec 2;19(12):e0312001. doi: 10.1371/journal.pone.0312001 (PMC11611106; doi:10.1371/journal.pone.0312001)
Supplement: S5 Table — (DOCX) [file pone.0312001.s005.docx]

**S5 Table. Risk of bias among included study**

| **Study** | **Year** | **Random sequence generation (selection bias)** | **Allocation conceclment (selection bias)** | **Blinding of participants and personnel (perfomance bias)** | **Blinding of outcome assessment (detection bias)** | **Incomplete outcome data (attrition bias)** | **Selective reporting (reporting bias)** | **Risk of Bias** |
| --- | --- | --- | --- | --- | --- | --- | --- | --- |
| Acosta | 2022 | Low risk | Low risk | Low risk | Low risk | Low risk | Low risk | High Risk |
| Bediang | 2018 | Low risk | Unclear risk | Low risk | Low risk | Low risk | Low risk | High Risk |
| Belknap | 2018 | Low risk | Low risk | High Risk | High Risk | Low risk | Low risk | High Risk |
| Browne | 2019 | Low risk | Low risk | Low risk | Low risk | Low risk | Low risk | Low risk |
| Burzynski | 2022 | Low risk | Low risk | Unclear risk | Unclear risk | Low risk | Low risk | High Risk |
| Cattamanchi | 2021 | Low risk | Unclear risk | Low risk | Low risk | Low risk | Low risk | Unclear risk |
| Doltu | 2021 | Low risk | High Risk | High Risk | High Risk | Unclear risk | Low risk | High Risk |
| Guo | 2019 | Low risk | Low risk | Low risk | Low risk | Low risk | Low risk | Low risk |
| Johnston | 2017 | Low risk | Unclear risk | Unclear risk | Unclear risk | Low risk | Low risk | High Risk |
| Louwagie | 2022 | Low risk | Low risk | Low risk | Unclear risk | Unclear risk | Low risk | High Risk |
| Manyazewal | 2022 | Low risk | Low risk | Low risk | Low risk | Low risk | Low risk | Low risk |
| Ravenscroft | 2020 | Low risk | Unclear risk | High Risk | High Risk | Low risk | Low risk | High Risk |
| Story | 2019 | Low risk | Low risk | Low risk | Low risk | Low risk | Low risk | Low risk |
